# Accuracy and comparison of sensor-based gait speed estimations under standardized and daily life conditions in children undergoing rehabilitation

**DOI:** 10.1186/s12984-022-01079-3

**Published:** 2022-10-04

**Authors:** Fabian Marcel Rast, Seraina Aschwanden, Charlotte Werner, László Demkó, Rob Labruyère

**Affiliations:** 1 Swiss Children’s Rehab, University Children’s Hospital Zurich, Affoltern am Albis, Switzerland; 2 Children’s Research Center, University Children’s Hospital of Zurich, University of Zurich, Zurich, Switzerland; 3 Rehabilitation Engineering Laboratory, Department of Health Sciences and Technology, ETH Zurich, Zurich, Switzerland; 4 Spinal Cord Injury Center, Balgrist University Hospital, Zurich, Switzerland

**Keywords:** Pediatric rehabilitation, Walking, Wearable inertial sensors, Data processing algorithm, Clinical assessments, Everyday life

## Abstract

**Background:**

Gait speed is a widely used outcome measure to assess the walking abilities of children undergoing rehabilitation. It is routinely determined during a walking test under standardized conditions, but it remains unclear whether these outcomes reflect the children's performance in daily life. An ankle-worn inertial sensor provides a usable opportunity to measure gait speed in the children's habitual environment. However, sensor-based gait speed estimations need to be accurate to allow for comparison of the children's gait speed between a test situation and daily life. Hence, the first aim of this study was to determine the measurement error of a novel algorithm that estimates gait speed based on data of a single ankle-worn inertial sensor in children undergoing rehabilitation. The second aim of this study was to compare the children’s gait speed between standardized and daily life conditions.

**Methods:**

Twenty-four children with walking impairments completed four walking tests at different speeds (standardized condition) and were monitored for one hour during leisure or school time (daily life condition). We determined accuracy by comparing sensor-based gait speed estimations with a reference method in both conditions. Eventually, we compared individual gait speeds between the two conditions.

**Results:**

The measurement error was 0.01 ± 0.07 m/s under the standardized and 0.04 ± 0.06 m/s under the daily life condition. Besides, the majority of children did not use the same speed during the test situation as in daily life.

**Conclusion:**

This study demonstrates an accurate method to measure children's gait speed during standardized walking tests and in the children's habitual environment after rehabilitation. It only requires a single ankle sensor, which potentially increases wearing time and data quality of measurements in daily life. We recommend placing the sensor on the less affected side, unless the child wears one orthosis. In this latter case, the sensor should be placed on the side with the orthosis. Moreover, this study showed that most children did not use the same speed in the two conditions, which encourages the use of wearable inertial sensors to assess the children's walking performance in their habitual environment following rehabilitation.

**Supplementary Information:**

The online version contains supplementary material available at 10.1186/s12984-022-01079-3.

## Background

Pediatric rehabilitation aims to foster functional independence in everyday life activities of children with congenital and acquired illnesses and injuries. For most families with children undergoing rehabilitation, improvements in self-care and mobility activities are prioritized [1], and thereby, most rehabilitation goals are chosen in the domain of walking [2]. Therefore, assessing gait-related outcomes is essential to tailor therapy to the families’ needs and monitor the children’s progress over time.

The 10-m walk test is the most widely used clinical assessment to determine gait speed in patients with neurological conditions [3]. This outcome often serves as a surrogate to assess the children’s overall walking abilities. Besides, increasing gait speed is essential since many children want to keep up with their peers [4]. The 10-m walk test is conducted under standardized conditions, and it is currently unclear whether children can translate their improvements into daily life after rehabilitation. To close this knowledge gap, appropriate measurement tools to assess the children’s gait speed in their habitual environment are needed.

Technological progress has made wearable inertial sensors small-sized, cost-effective, energy-efficient, and thus ideal for conducting long-term measurements in daily life [5]. Hence, many algorithms with different approaches were developed to estimate gait speed based on sensor data [6]. To be sensitive, these algorithms must provide accurate gait speed estimations with a measurement error smaller than the minimally important change (MIC) for children undergoing rehabilitation. The MIC has been well investigated in adults with various pathologies and lies between 0.10 m/s and 0.20 m/s [7]. There is some evidence that the MIC for children lies within a similar range [8, 9], and we used a MIC of 0.10 m/s as a benchmark to evaluate the algorithm’s measurement error in this study.

Children with walking impairments present complex gait deviations [10], and algorithms should be validated specifically in this population. Based on the authors’ knowledge, only two studies investigated the measurement error of sensor-based gait speed estimations in children with walking impairments [11, 12]. These studies validated three different sensor configurations and their underlying algorithms. Two algorithms revealed measurement errors smaller than the MIC. However, the usability of the related sensor configurations for long-term measurements in daily life is questionable. The first algorithm uses data of a foot-worn sensor [11, 12], but children often change or take off their footwear during daily life. The second algorithm requires sensors placed on each thigh and shank [12], and the need to wear four sensors simultaneously might decrease children's compliance. The third algorithm uses a single ankle-worn sensor [12], but the algorithm resulted in a measurement error larger than the MIC. Since we believe that this sensor configuration is the preferred choice in terms of usability, an improved algorithm with accurate gait speed estimations based on a single ankle-worn sensor is needed.

Consequently, the first aim of this study was to determine the measurement error of an improved algorithm that estimates gait speed based on data of a single ankle-worn inertial sensor in children undergoing rehabilitation. We investigated on which ankle the sensor needs to be placed to optimize accuracy and whether the measurement error is smaller than the MIC. The second aim of this study was to compare children’s gait speed between standardized and daily life-like conditions.

## Methods

### Participants and recruitment

A convenience sample of 24 children and adolescents was recruited at the Swiss Children’s Rehab of the University Children’s Hospital Zurich, Switzerland. These children were able to walk household distances and had the rehabilitation goal to improve their walking abilities. Further inclusion criteria were: age between 4 and 20 years, cognitive abilities to follow instructions, and informed consent to participate in the study. Exclusion criteria were exacerbating pain during walking and open skin lesions at the ankle. The local ethics committee has classified the study as not requiring approval (BASEC Nr.: Req-2017-00958).

### Procedure and equipment

**Fig. 1:**
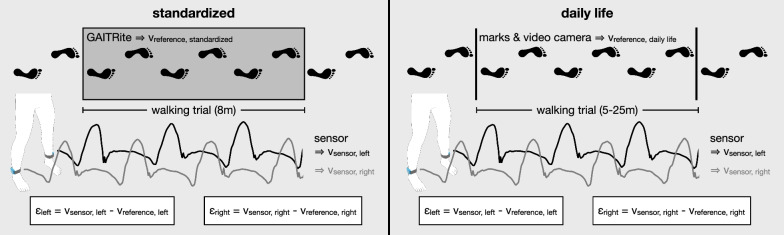
Procedure to determine the measurement error ε of both sides and in both conditions

Participants were equipped with two wearable inertial sensors [13]. The sensors were placed above the lateral malleoli with corresponding hook-and-loop straps (Fig. 1). The 3-axis accelerometer and gyroscope signals were used for data processing. Besides, participants were equipped with a video camera (YI 4 K Action Camera, YI Technology, Shanghai, China). The camera was fixed to the chest with a harness, facing downward to take video recordings of the feet. The sampling rate of both devices was set to 50 Hz, and timestamps were synchronized by quickly rotating one sensor in front of the camera.

To study the gait speed in a standardized condition, participants completed four 10-m walk tests at different speeds. They were instructed to walk at their self-selected speed during the first two trials. Then, they were asked to walk at a slower speed, and for the last trial, they had to walk as fast as they safely could without running. This setting was chosen to reflect the variability of gait speeds in daily life and challenge the algorithm with slow and fast walking trials. The GAITRite (CIR Systems, Franklin, USA), an 8 m pressure-sensitive walkway, was used as a reference to determine the measurement error of the sensor-based gait speed estimation. The GAITRite proprietary software derived the average gait speed (v_reference, standardized_) for each participant and walking trial.

In the daily life condition, participants wore the sensor system for one hour while having leisure or school time at the clinic. During this period, the participants walked between the research department, their room, and the school, all located in the same building. The number of walking trials depended on their schedule and self-selected activities, while they interacted with other patients and the staff as usual. As a reference, we placed marks on the floor of straight hallways (5–25 m apart) distributed across the clinic and determined the durations participants needed to cover those distances from the chest video recordings to calculate the actual gait speed v_reference, daily life_. Uninterrupted walking trials between two marks were analyzed. This condition was chosen to challenge the algorithm with non-standardized walking trials and to determine the children's daily gait speed at the clinic.

### Data processing

Walking trials were defined by stepping on and off the GAITRite under standardized conditions and stepping over a mark in daily life. These time points were determined from the video recordings. The sensor-based walking speed was estimated from the ankle-worn sensors, by applying an algorithm originally developed for the spinal cord injured population [14]. In brief, the algorithm derives an adaptive threshold from the main frequency component of the gyroscope data, which corresponds to the cadence of walking. This threshold determines a window size in which the algorithm searches for local peaks in the gyroscope and acceleration data to identify specific gait events (e.g., initial contact, final contact, mid-swing, and mid-stance). Then, the algorithm obtains the stride duration, defined as the time between two successive initial contacts. After preprocessing the acceleration data to remove the gravity component, the pure movement acceleration is integrated twice to obtain the 3D sensor trajectory. A part of this double integration process is a modified “zero-velocity” update, based on an estimated ankle velocity during mid-stance, to reduce the effect of drift in the data. Finally, the stride length is retrieved from the 3D sensor trajectory, and the walking speed is estimated as the stride length divided by the corresponding stride duration. The algorithm was applied to the sensor data of all walking trials, for the left and right sides separately, to calculate the average gait speed v_sensor, standardized_ and v_sensor, daily life_. The procedure to determine gait speeds and measurement error is illustrated in Fig. 1.

### Statistical analysis

The absolute error ε_absolute_ =|v_sensor_ - v_reference_| was used to analyze which side reveals more accurate gait speed estimations. Participants were divided into two groups. Participants with similar impairments of their legs were allocated to the symmetrical group. In contrast, the asymmetrical group comprised participants with a unilateral impairment or with a more affected leg. For each walking trial, the difference of ε_absolute_ between the left and right sides for the symmetrical group and between the more and less affected sides for the asymmetrical group was calculated. These differences were non-normally distributed. Consequently, the median was calculated for each participant, including all walking trials of both conditions. The Wilcoxon signed-rank test was applied to determine whether the measurement error differed between the two sides.

**Fig. 2:**
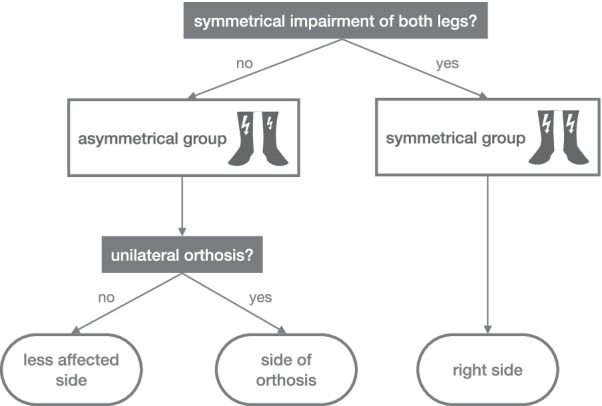
Decision tree for the placement of a single ankle sensor

Further analyses were conducted on single ankle sensors, taking only the side selected based on the absolute error differences introduced above. The selection process is depicted in Fig. 2 and justified in the discussion section. The 95% limits of agreement (LoA) between the sensor-based gait speed estimations and the reference values were calculated separately for both conditions [15]. Then, the smallest detectable change (SDC) was estimated by multiplying the LoA with $\sqrt{2}$ [16] and was then compared to the MIC (0.1 m/s). To calculate the measurement error of the average gait speed over several walking trials, the standard deviation of the measurement error was divided by the square root of the number of walking trials. This provides a method to determine how many walking trials are required to reach SDCs smaller than the MIC.

For each participant, the average gait speed of all walking trials during daily life was compared to the average gait speed of the two 10-m walk tests with self-selected speed. The number of participants who walked faster, slower, and equally fast in daily life than the standardized condition was counted. Walking speeds were considered to be equal if their difference was less than the MIC.

## Results

Seven girls and 17 boys (12.3 ± 3.3 years) with various medical diagnoses completed the study protocol. The participants' diagnoses, their individual walking abilities, measured with the walking scale of the Gillette Functional Assessment Questionnaire (GFAQ) [17], and their use of walking aids and orthoses are listed in Table 1. The GFAQ describes the children's typical walking abilities and ranged between 5 (child walks more than 15–50 feet but only inside at home or school (walks for household distances)) and 10 (walks, runs, and climbs on level and uneven terrain without difficulty or assistance). The median GFAQ was 8 with an interquartile range of 1.8. Thirteen participants required walking aids, orthoses, or assistance from another person, while the remaining 11 participants walked without assistance.

**Table 1:** Patients' walking abilities and side comparison of the absolute measurement error

Symmetrical impairment						
ID	GFAQ	Diagnosis	Walking aids	Orthoses	Median difference^1^ (m/s)	p-value
2	10	Hereditary ataxia	None	None	− 0.02	0.27
8	10	Encephalopathy	None	None	0.00	0.54
9	7	Neoplasms	Assistance from another person	None	0.01	0.05
11	9	Dissociative movement disorder	None	None	0.00	0.59
12	8	Traumatic brain injury	None	None	− 0.01	0.11
13	5	Cardiogenic shock	None	Foot lifter (both sides)	0.00	0.84
14	7	Genetic disorder	Posterior walker	Ankle–foot (both sides)	0.02	0.13
20	8	Dissociative movement disorder	Crutches (both sides)	None	− 0.01	0.57
22	6	Traumatic brain injury	Posterior walker	None	− 0.03	**0.03**
24	5	cerebral palsy	Posterior walker	Ankle–foot (both sides)	0.00	0.64

Asymmetrical impairment						
ID	GFAQ	Diagnosis	Walking aids	Orthoses	Median difference^1^ (m/s)	p-value
1	8	Traumatic brain injury	None	None	− 0.03	**0.00**
3	8	Traumatic spinal cord injury	None	None	− 0.02	0.20
4	6	Neoplasms	Posterior walker	None	− 0.01	0.43
5	10	Traumatic brain injury	None	None	0.00	0.67
6	8	Neoplasms	Crutch (right side)	None	**0.21**	**0.01**
7	6	Cerebral palsy	Posterior walker	Ankle–foot (both sides)	0.01	0.69
10	9	Cerebrovascular disease	None	None	0.01	0.64
15	8	Polytrauma	Crutches (both sides)	Ankle–foot (left side)	− 0.05	**0.00**
16	8	Cerebral palsy	Crutches (both sides)	Ankle–foot (left side)	− 0.01	0.94
17	8	Cerebral palsy	None	None	− 0.01	0.30
18	7	Polyneuropathy	None	Foot lifter (left side)	− **0.09**	**0.01**
19	6	Cerebral palsy	None	Ankle–foot (both sides)	− 0.02	0.25
21	8	Traumatic Brain injury	None	None	0.00	0.72
23	8	Cerebral palsy	None	None	0.00	1.00

^1^The median difference of the absolute measurement error: left - right side in the symmetrical group and more affected - less affected side in the asymmetrical group. Large and statistically significant differences are indicated with bold numbers

*GFAQ*  Walking scale of the Gillette Functional Assessment Questionnaire

Missing values occurred at the fast walking trial of participant 17 and the first walking trial with the self-selected speed of participant 23 due to failure of the GAITRite. In daily life, the number of performed walking trials varied and ranged from 1 to 13. On average, walking trials contained 15 steps during the standardized condition and 22 steps in daily life. This resulted in an overall dataset of 230 walking trials and 4360 steps.

For each participant, the difference of measurement errors between sides and the statistical test results are shown in Table 1. Children with symmetrical impairment revealed either small or non-significant differences between the measurement error of the left and right sides. This indicates that there is no clear favorable side in these patients, and we arbitrarily decided to continue the analysis with the data of the right side. However, two children with an asymmetrical impairment revealed large and significant differences between the measurement errors of the more and less affected sides. Thus, we decided to continue the analysis with the side revealing smaller measurement errors in this group. This was usually the less-affected side unless children had worn only one orthosis. In this case, it was the side with the orthosis (Fig. 2).

**Fig. 3:**
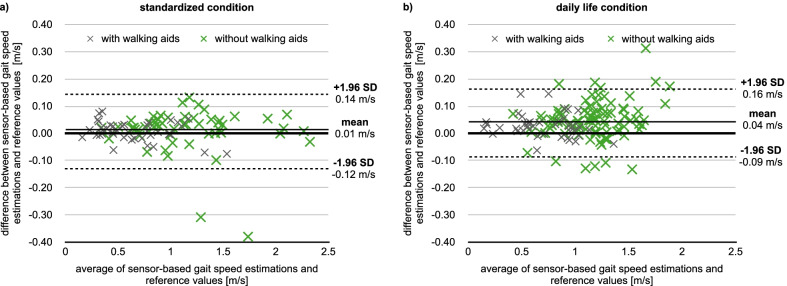
Bland–Altman plots of the gait speed estimations in standardized (**a**) and daily life conditions (**b**). Gray data points correspond to participants walking with aids, while green data points correspond to those walking without aids

The measurement errors of gait speed estimations based on a single ankle sensor were 0.01 ± 0.07 m/s (LoA: ± 0.13 m/s) under the standardized, and 0.04 ± 0.06 m/s (LoA: ± 0.12 m/s) under the daily life condition, respectively, and are visualized in Fig. 3. The SDC was 0.19 m/s for the standardized and 0.18 m/s for the daily life condition. Therefore, the measurement error of single walking trials is too large to detect MIC. However, averaging the gait speed estimations of four walking trials would be accurate enough to detect MICs in both conditions. In addition to the standard analysis, we also normalized the measurement error by gait speed, which is illustrated in Additional file 1. The LoA were ± 14% for both conditions.

**Fig. 4:**
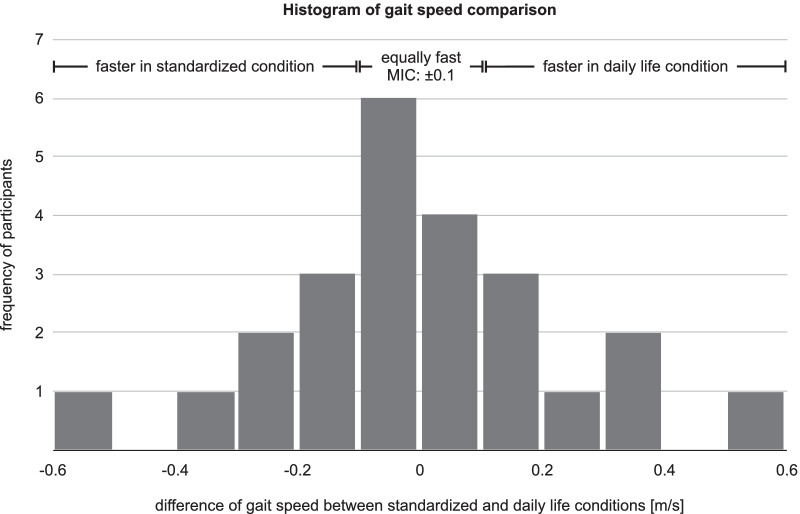
Histogram of the individual gait speed differences between the standardized and the daily life condition. *MIC* minimal important change

Individual differences in gait speed between the two conditions are shown in Fig. 4. Seven children walked faster during the 10-m walk test (by at least the MIC), while also seven children walked faster in daily life. The remaining ten participants walked equally fast in both conditions.

## Discussion

In this study, we determined the accuracy of a novel algorithm that estimates gait speed based on data of a single ankle-worn inertial sensor in a heterogeneous sample of children and adolescents undergoing rehabilitation. We investigated on which ankle the sensor should be placed to optimize accuracy and how many walking trials need to be recorded to get accurate gait speed estimations. Furthermore, we explored whether children use different self-selected gait speeds in standardized and daily life-like conditions.

In most participants, we observed no difference in measurement errors of gait speed estimations based on data of the left and right ankles or the less and more affected sides. However, two participants had large and significant differences between the measurement errors of the more and less affected sides. Participant 6 has a hemiparesis and revealed smaller measurement errors on the less affected side. The detection of mid-stance failed due to excessive toe walking on the more affected side. This resulted in wrong boundary conditions for the double integration of the acceleration signal and an overestimation of the stride length. In contrast, participant 18 showed smaller measurement errors on the more affected side. This participant has a bilateral peroneal nerve paralysis that is more severe on the left side. Wearing a foot lifter orthosis on this side resulted in a heel strike gait pattern on the more affected side, which has been observed in previous studies and with other orthoses, too [18]. This gait pattern improved the detection of gait events and most likely explains the smaller measurement errors. Similar results were observed in participants 15 and 16 who wore the orthosis on just one side. Consequently, we recommend placing the sensor as shown in the decision tree in Fig. 2. Still, this recommendation is mainly based on the results of two participants and has to be confirmed with the results of future studies.

As seen in Fig. 3, the algorithm overestimated gait speed slightly, and this overestimation was larger in daily life than in the standardized condition. This difference can be explained by the chosen reference method of the daily life condition. We assumed that children walked straight between two consecutive marks on the floor. However, as confirmed by the video recordings, this was not always the case and led to underestimated gait speeds in the reference values. Hence, we expect that the true systematic error in daily life is smaller than reported in this study and comparable to that of the standardized condition. This justifies why we did not correct for systematic errors when comparing the gait speed between standardized and daily life conditions.

Except for two outliers in the standardized condition, the random error was larger in daily life compared to the standardized condition. We believe that the larger variance of walking trials in daily life can explain the larger error observed in Fig. 3b. This higher variance could be due to changes in the walking direction (e.g., due to obstacles), interactions with others, etc. Both outliers occurred during the fast walking trial of two participants and could be explained by data loss due to short stride durations and the too low sampling rate of 50 Hz to capture the actual movement.

The Bland–Altman plots in Fig. 3 allow for a visual investigation of the relationship between gait speed and measurement error. Even though the error seems to be slightly higher for fast walking speeds in the daily life condition, we could not observe a strong dependence of measurement error on gait speed. A statistical analysis of this relationship would not be valid with our current study design since gait speed varied between participants. Therefore, a significant correlation could also be caused by slow or fast walking individuals presenting a particular gait pattern, leading to relatively small or large measurement errors. Besides, we colored the data points in Fig. 3 to visually investigate the influence of walking aids on the measurement error. Gray data points correspond to participants walking with aids, while green data points correspond to those walking without aids. For the latter, the measurement error seems to be slightly higher in daily life. However, they also walked at faster speeds. Again, our study design does not allow for disentangling the influence of speed and assistive devices on the measurement error. Therefore, larger studies with balanced designs are needed to investigate the relationship between measurement error, gait speed, assistive devices, and gait patterns.

The LoA in this study (0.13 m/s) are narrower than that of a similar study (0.21–0.26 m/s) investigating the accuracy of ankle sensor-based gait speed estimations in children with cerebral palsy [12]. Even though they reported more accurate results using the foot sensor data (0.12–0.24 m/s) compared to that of the ankle position, our method with a single ankle sensor provides a level of accuracy comparable to these higher accuracy values. However, the comparison is difficult since the previous study reported measurement errors of individual strides instead of walking trials of several steps, and averaging the gait speed over multiple strides might lead to more accurate results.

The majority of participants did not choose the same walking speed during the 10-m walk test and in daily life at the clinic. Half of these participants walked faster, and the other half slower during the test. This is in line with previous research investigating the gait speed of children with cerebral palsy in both conditions, observing a highly heterogeneous behavior in terms of gait speed selection [19]. Children's behavior seems to be different from that of adults with neuromotor impairments [20] or healthy adults [21], who mostly walk faster during the standardized tests. Nevertheless, all of these studies emphasize that gait speed in a test situation and that in daily life can be significantly different. This encourages the use of wearable inertial sensors to assess the children's walking performance after rehabilitation in their habitual environment at home and school. Furthermore, future research could also identify personal and environmental factors that explain why some children walk faster and some slower in the test than in daily life.

The validation protocol was a key component of our study. While previous studies determined the accuracy of the related algorithms by walking trials of standardized conditions and self-selected speed [11, 12], we also instructed our participants to additionally walk at a slower and faster pace, and further, implemented a method to estimate the walking speed of daily life by video analysis and used it as a reference. This protocol added variance to the collected data and reflected real-world data more comprehensively, challenging the algorithm, and leading to a more valid estimation of the measurement error. Still, the daily life condition in our study comprised only straight and flat hallways inside the clinic, while the children's habitual environment might also include curvy walking paths and uneven surfaces. This increase in variability might lead to larger measurement errors. Moreover, our algorithm needs to be combined with a walking detection algorithm to be applicable to unlabeled daily life data. This might further increase the measurement error of gait speed estimations since non-walking episodes falsely labeled as walking could lead to abnormal gait speed estimations. Besides, using gait speed as a surrogate to detect changes in the children's overall walking capacity due to an intervention depends not only on the measurement error but also on the children's behavior during the measurement. Therefore, future studies should address the test–retest reliability in the standardized condition and the between-day reliability in daily life to determine the SDC considering the measurement error and other sources of variance such as the child's motivation or daily activities.

## Conclusions

We evaluated a novel algorithm that determines gait speed based on a single ankle-worn inertial sensor in children undergoing rehabilitation. The accuracy was comparable to previously reported algorithms and superior in terms of the number and position of required sensors. This is a clear advantage that potentially increases the wearing time and thus data quality when monitoring children's gait over multiple days. The comparison of the children's self-selected gait speed between the standardized test and in daily life showed that the majority of children did not use the same speed in the two conditions, which encourages the use of wearable inertial sensors to assess the children's walking performance in their habitual environment following rehabilitation.

## Supplementary Information

**Additional file 1.** Bland–Altman plots of the gait speed estimations in standardized (a) and daily life conditions (b). The measurement error has been normalized by gait speed. Gray data points correspond to participants walking with aids, while green data points correspond to those walking without aids.

## Data Availability

The datasets used and analyzed during the current study are available from the corresponding author on reasonable request.

## Abbreviations

GFAQ: Gillette Functional Assessment Questionnaire
LoA: Limits of agreement
MIC: Minimally important change
SDC: Smallest detectable change
