# Supplementary figures and images for: Accuracy and comparison of sensor-based gait speed estimations under standardized and daily life conditions in children undergoing rehabilitation
Source: J Neuroeng Rehabil. 2022 Oct 4;19:105. doi: 10.1186/s12984-022-01079-3 (PMC9531434; doi:10.1186/s12984-022-01079-3)

a)

standardized condition

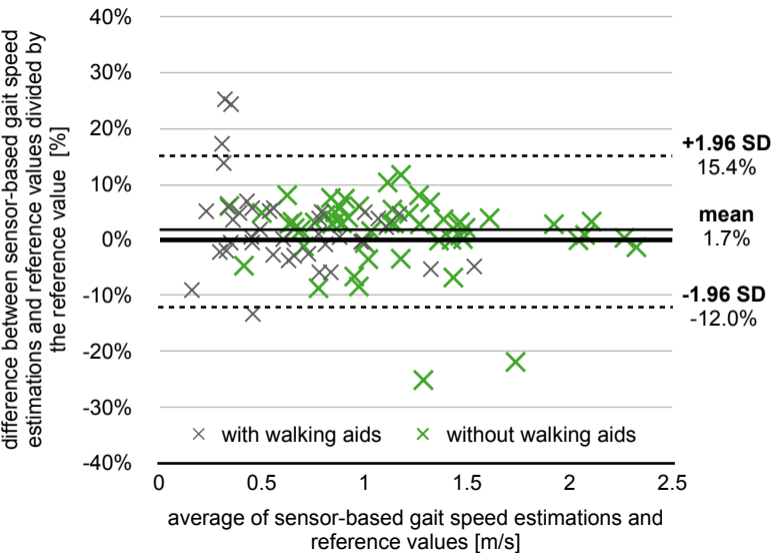

b)

daily life condition

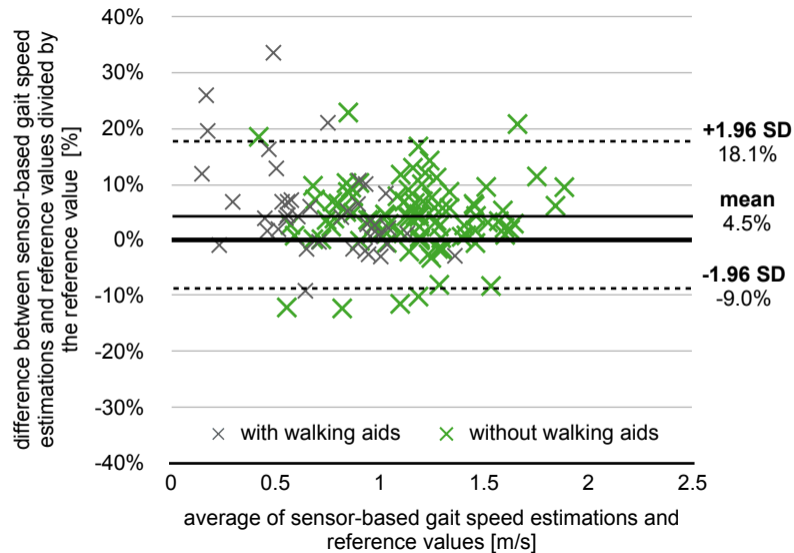

Supplement: Supplementary file 1 — Additional file 1. Bland–Altman plots of the gait speed estimations in standardized (a) and daily life conditions (b). The measurement error has been normalized by gait speed. Gray data points correspond to participants walking with aids, while green data points correspond to those walking without aids. [file 12984_2022_1079_MOESM1_ESM.pdf]
